# Machine learning-based prediction of carotid intima–media thickness progression: a three-year prospective cohort study

**DOI:** 10.3389/fmed.2025.1593662

**Published:** 2025-06-12

**Authors:** An Zhou, Kui Chen, Yonghui Wei, Qu Ye, Yuanming Xiao, Rong Shi, Jiangang Wang, Wei-Dong Li

**Affiliations:** ^1^Department of Genetics, College of Basic Medical Sciences, Tianjin Medical University, Tianjin, China; ^2^Health Management Medical Center, Third Xiangya Hospital, Central South University, Changsha, China; ^3^State Key Laboratory of Ultrasound in Medicine and Engineering, College of Biomedical Engineering, Chongqing Medical University, Chongqing, China; ^4^Department of Clinical Laboratory, Peking University First Hospital, Beijing, China

**Keywords:** carotid intima–media thickness (CIMT), machine learning, atherosclerosis progression, risk prediction, cardiovascular prevention

## Abstract

**Background:**

Early detection of subclinical atherosclerosis progression is crucial for preventing atherosclerotic cardiovascular disease (ASCVD). Carotid intima–media thickness (CIMT) is a recognized surrogate marker for atherosclerosis, but accurate prediction of its progression remains challenging. This study aimed to develop and validate machine learning models for predicting CIMT progression via routine clinical biomarkers.

**Methods:**

In this three-year prospective cohort study, we analyzed data from 904 participants from the Third Xiangya Hospital of Central South University Health Examination Cohort who underwent three consecutive annual CIMT measurements. The participants were categorized into CIMT thickening and nonthickening groups on the basis of a final CIMT ≥1.0 mm or an increase ≥0.1 mm across consecutive measurements. We evaluated seven machine learning algorithms: logistic regression, random forest, XGBoost, support vector machine (SVM), elastic net, decision tree, and neural network. Model performance was assessed through discrimination (AUC, sensitivity, specificity) and calibration metrics, with Platt scaling applied to optimize probability estimates. Clinical utility was evaluated through decision curve analysis.

**Results:**

Compared with the more complex algorithms, the elastic net model demonstrated superior performance (AUC 0.754). Baseline CIMT, absolute monocyte count, sex, age, and LDL-C were identified as the most influential predictors. After Platt scaling, the calibration improved significantly across all the models. Decision curve analysis revealed a positive net benefit across a wide threshold range (0.01–0.5). On the basis of calibrated probabilities, we developed a three-tier risk stratification framework that identified distinct groups with progressively higher event rates: medium-risk (13.9%), high-risk (50.0%), and very-high-risk (60.0%). Subgroup analysis revealed better predictive performance in younger participants (<50 years), those with lower baseline CIMT (<0.8 mm), and females.

**Conclusion:**

Machine learning approaches, particularly the elastic net model, can effectively identify individuals at high risk for CIMT progression via routine clinical biomarkers. The superior performance of simpler models suggests predominantly linear relationships between predictors and CIMT progression. Following appropriate calibration, the model demonstrated strong clinical utility across diverse decision thresholds, supporting a stratified approach to atherosclerosis prevention.

## 1 Introduction

Atherosclerotic cardiovascular disease (ASCVD) remains the leading cause of mortality and morbidity worldwide, with atherosclerosis as its primary pathophysiological mechanism (1). Early detection and intervention of subclinical atherosclerosis represent key strategies for reducing the global burden of ASCVD. Carotid intima–media thickness (CIMT), measured by ultrasonography, has emerged as a recognized surrogate marker for atherosclerosis and a powerful predictor of future cardiovascular events (2).

CIMT measurement offers multiple advantages as a clinical tool: it is noninvasive, relatively cost-effective, widely available, and highly reproducible when standardized protocols are followed (3, 4). Numerous longitudinal studies have confirmed that increased CIMT is independently associated with elevated risks of myocardial infarction, stroke, and cardiovascular mortality (5). Moreover, some studies suggest that baseline CIMT measurements provide valuable prognostic information for cardiovascular risk prediction (6, 7).

Despite these advantages, the clinical application of CIMT remains limited by challenges in predicting individual progression over time. Current approaches typically rely on established risk factors and scoring systems designed to predict cardiovascular events rather than CIMT progression (8). These methods generally demonstrate moderate predictive performance and fail to capture complex nonlinear relationships between risk factors and subclinical atherosclerosis progression (9). Consequently, more accurate predictive tools are urgently needed to identify individuals at highest risk for CIMT progression who might benefit most from intensified preventive interventions (10).

Machine learning (ML) methods offer promising solutions to these challenges through their ability to model complex nonlinear relationships and interactions among multiple predictors (11). Some studies suggest that ML algorithms have the potential to improve cardiovascular risk prediction compared with traditional statistical methods (12–14). However, most ML applications in cardiovascular medicine have focused on predicting clinical events rather than subclinical markers of disease progression (15). Although it is a valuable predictor of ASCVD, no ML-related studies exist.

In this three-year prospective cohort study, we aimed to develop and validate ML models for predicting CIMT progression via readily available clinical and laboratory parameters from the Xiangya Third Hospital of Central South University Health Examination Cohort. We evaluated multiple ML algorithms, including logistic regression, random forest, XGBoost, support vector machine, elastic net, decision tree, and neural network methods. We assessed model performance through comprehensive metrics of discrimination and calibration and applied Platt scaling to optimize probability estimates. Finally, we evaluated the potential clinical utility of these models at different threshold probabilities through decision curve analysis.

By establishing accurate CIMT progression prediction models, this study aims to facilitate early identification of individuals at high risk for atherosclerosis, allowing for targeted preventive interventions before the development of clinical cardiovascular disease by extending the prediction window for ASCVD. This approach aligns with the evolving paradigm of precision medicine and may contribute to more efficient allocation of cardiovascular prevention resources.

## 2 Materials and methods

### 2.1 Study population

The present study utilized biochemical and hematological indices from 128,938 individuals enrolled in the “Third Xiangya Hospital of Central South University Health Examination Cohort” established in 2015. Following preliminary screening, 54,212 records were included in the cohort, while the remainder were excluded because of incomplete documentation. This cohort underwent annual health examinations, with 31,158 individuals enrolled between 2015 and 2023. The cohort encompasses not only biochemical parameters but also carotid intima–media thickness (CIMT) measurements at four anatomical locations (left/right carotid bifurcation and distal left/right common carotid artery). Our predictive model was developed on the basis of the mean CIMT values across these four locations.

### 2.2 Patient selection

**Figure 1:**
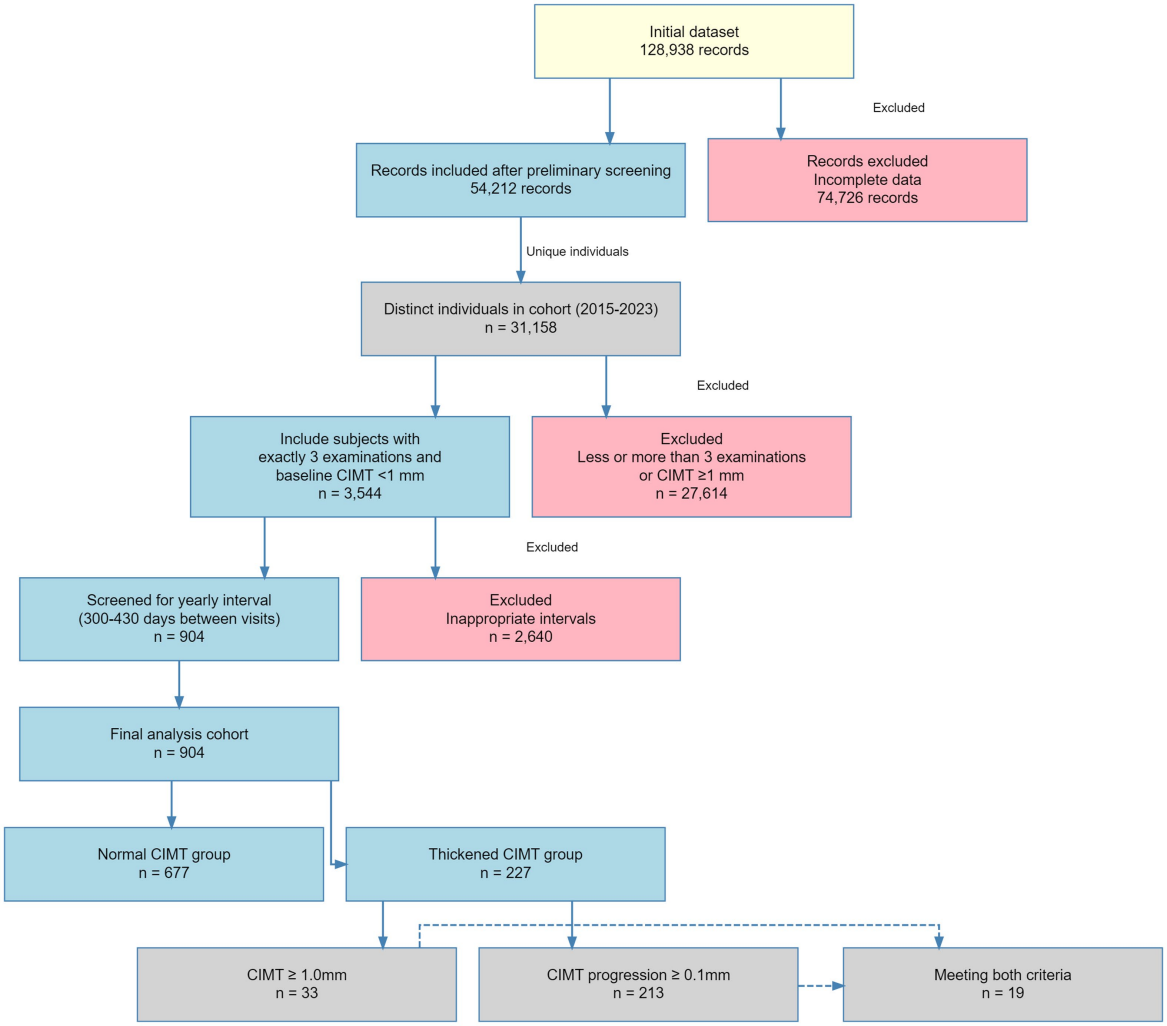
Study cohort selection process for CIMT progression analysis.

From the initial database of 31,158 participants, we established a longitudinal cohort with regular follow-up intervals to assess carotid intima–media thickness (CIMT) progression. We first screened patients who completed three independent CIMT measurements during health examinations and had baseline CIMT values <1 mm (*n* = 3,544). To ensure standardized follow-up intervals, only participants with adjacent examinations spaced 300–430 days apart (approximately annual intervals) were included (*n* = 904). This time window allows reasonable scheduling flexibility while maintaining the periodicity of annual assessments. Among these 904 participants, in accordance with clinical guidelines and previous research, we divided the population into CIMT thickening and nonthickening groups according to the following criteria: a final examination of CIMT ≥1.0 mm (3, 16–18) or increase ≥0.1 mm (19–21) across consecutive measurements (Figure 1).

After completing the subject screening, we first evaluated the proportion of missing values for all the variables. Variables with >20% missing data were excluded. Correlation analysis was performed on retained variables to identify multicollinearity, eliminating the clinically less significant variable from highly correlated variable pairs (correlation coefficient >0.7).

For the remaining variables, missing data were imputed via predictive mean matching (PMM), generating five imputed datasets (*m* = 5, maxit = 50), with the first complete dataset selected for subsequent analysis. Near-zero variance predictors were identified and removed via the nearZeroVar function from the caret package.

Feature selection was conducted via the random forest-based Boruta algorithm, which identifies statistically significant variables for classification tasks through the shadow attribute method. The algorithm runs for 100 iterations (maxRuns = 100), retaining variables confirmed as “important” by Boruta and “tentative” variables. Additionally, age and sex were forcibly included as clinically important variables regardless of Boruta analysis results.

### 2.3 Model development and performance evaluation

The dataset was divided into training and testing sets at a 7:3 ratio via stratified sampling to maintain a consistent class distribution. To address class imbalance in the training set, a mixed sampling strategy from the ROSE package was employed (method = “both,” *p* = 0.5), which simultaneously oversamples the minority class and undersamples the majority class. All the models were optimized through 5-fold cross-validation (repeated 3 times), with the area under the receiver operating characteristic curve (AUC) as the primary metric for model selection during cross-validation.

We developed models via seven machine learning algorithms: logistic regression, random forest, XGBoost, support vector machine with a radial basis function (SVM) kernel, elastic net, decision tree, and neural network.

To validate model performance, we assessed the following metrics: area under the curve (AUC), sensitivity, specificity, positive predictive value (PPV), negative predictive value (NPV), *F*_1_-score, expected calibration error (ECE), Brier score, and log loss.

Model calibration was performed via Platt scaling, which involves fitting a logistic regression to transform the original model outputs. We tested three regularization methods (ridge L2, lasso L1, and elastic net) combined with stratified k-fold cross-validation for calibration model development. Calibration performance was assessed via the expected calibration error (ECE), Brier score, and log-loss metrics. Calibration curves were generated to visually evaluate the alignment between the predicted probabilities and actual outcomes before and after Platt scaling.

To evaluate model stability and data efficiency, we created learning curves by training models on increasing fractions (5, 10, 20, 50, and 100%) of the training dataset. For each fraction, we performed five iterations and calculated the mean AUC and standard deviation to assess performance stability across different training data volumes.

For subgroup analysis, we stratified the test set by age (≤35 years, 35–50 years, >50 years), sex (male, female), and baseline CIMT level (low: <0.6 mm, medium: 0.6–0.8 mm, high: >0.8 mm). Model performance and calibration effectiveness were evaluated separately for each subgroup via the same metrics applied to the overall population. This analysis helped assess whether model performance remained consistent across different demographic and clinical subgroups.

For feature importance analysis, we compared the coefficient magnitudes and significance from both elastic net and logistic regression models to provide comprehensive insights into predictor relevance. This comparative approach allowed for more robust identification of key predictors for CIMT thickening.

Finally, we conducted decision curve analysis (DCA) using the calibrated models. DCA estimates the net benefit of using prediction models to guide clinical decisions at different threshold probabilities. The DCA curve of the best-performing model was compared with two default strategies: “treat all” and “treat none.” This analysis helps identify the range of threshold probabilities where the model provides clinical value beyond these baseline strategies.

The optimal thresholds were determined via Youden index analysis, which identifies the point that maximizes the sum of sensitivity and specificity. On the basis of the DCA results and clinical considerations, we developed a risk stratification approach to classify patients into risk categories (medium, high, and very high risk) with corresponding intervention recommendations. Risk thresholds were determined on the basis of a combination of the Youden index, maximum net benefit point, and clinically significant event rates.

The DCA curve of the best-performing model was compared with two default strategies: “treat all” and “treat none” (22). This analysis helps identify the range of threshold probabilities where the model provides clinical value beyond these baseline strategies (23). In clinical decision analysis, “treat all” and “treat none” represent two extreme baseline strategies used as reference benchmarks to measure the clinical utility of prediction models: treating all ensures coverage of all patients needing treatment but leads to overtreatment (high false positives); treating none completely avoids overtreatment but misses all patients requiring treatment (high false negatives) (24). Through decision curve analysis, if a model’s net benefit curve exceeds both baselines within a specific threshold range, it indicates that selective treatment based on model predictions better balances treatment benefits and risks (25), delineating the clinical value interval for practical model application (26).

### 2.4 Statistical analysis

All analyses were conducted via R version 4.4.2. Statistical significance was set at *p* < 0.05.

## 3 Results

### 3.1 Baseline characteristics

On the basis of our thickening criteria (final examination CIMT ≥1.0 mm or increase ≥0.1 mm across consecutive measurements), 904 individuals from the “Third Xiangya Hospital of Central South University Health Examination Cohort” of 31,158 participants were included for model development (Figure 1).

No significant differences in age (42.0 vs. 43.0 years, *p* = 0.119), sex distribution (male: 63.1% vs. 68.3%, *p* = 0.181), or BMI (24.20 vs. 24.12 kg/m^2^, *p* = 0.568) were detected between the nonthickened and thickened groups. Blood pressure parameters were comparable between the groups: systolic pressure (122.0 vs. 121.0 mmHg, *p* = 0.919) and diastolic pressure (75.0 vs. 76.0 mmHg, *p* = 0.324). Lipid metabolism indices were not significantly different: total cholesterol (4.93 vs. 5.01 mmol/L, *p* = 0.319), triglycerides (1.40 vs. 1.44 mmol/L, *p* = 0.292), HDL-C (1.26 vs. 1.29 mmol/L, *p* = 0.625), and LDL-C (2.87 vs. 2.88 mmol/L, *p* = 0.386). The white blood cell count (6.09 vs. 5.95 × 10^9^/L, *p* = 0.916) and absolute monocyte count (0.36 vs. 0.38 × 10^9^/L, *p* = 0.140) were similarly distributed between the groups.

The most notable difference between the groups was the baseline CIMT: 0.75 mm (IQR: 0.65–0.80) in the nonthickened group versus 0.65 mm (IQR: 0.60–0.75) in the thickened group (*p* < 0.001, SMD = 0.509). These findings suggest that individuals with lower baseline CIMT may be overlooked by conventional risk assessments despite having higher actual progression risk. The absence of differences in traditional risk factors (e.g., age, lipid profiles) between the two groups may indicate limited predictive performance of these factors for CIMT progression in populations with normal baseline CIMT (Supplementary material 1).

### 3.2 Feature selection

**Figure 2:**
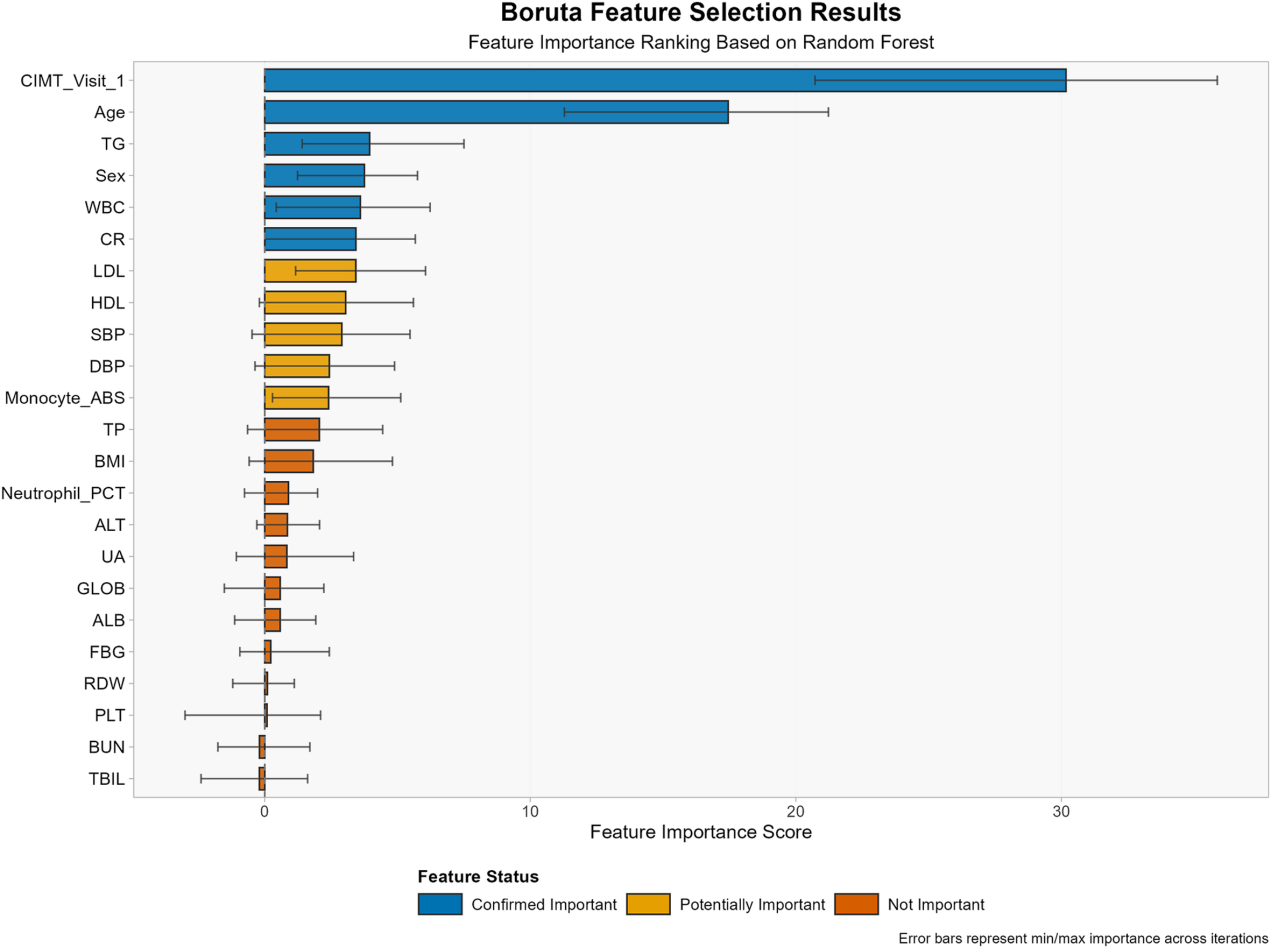
Feature selection results using the Boruta algorithm for CIMT thickening prediction.

Through the Boruta algorithm, we screened all 47 features (Figure 2). In terms of the calculated *Z* values, SBP, DBP, TG, HDL, LDL, CR, WBC, Monocyte_ABS, sex, age, and CIMT visit 1 were identified as variables closely associated with CIMT thickening.

### 3.3 Assessment of dataset covariate shift

**Figure 3:**
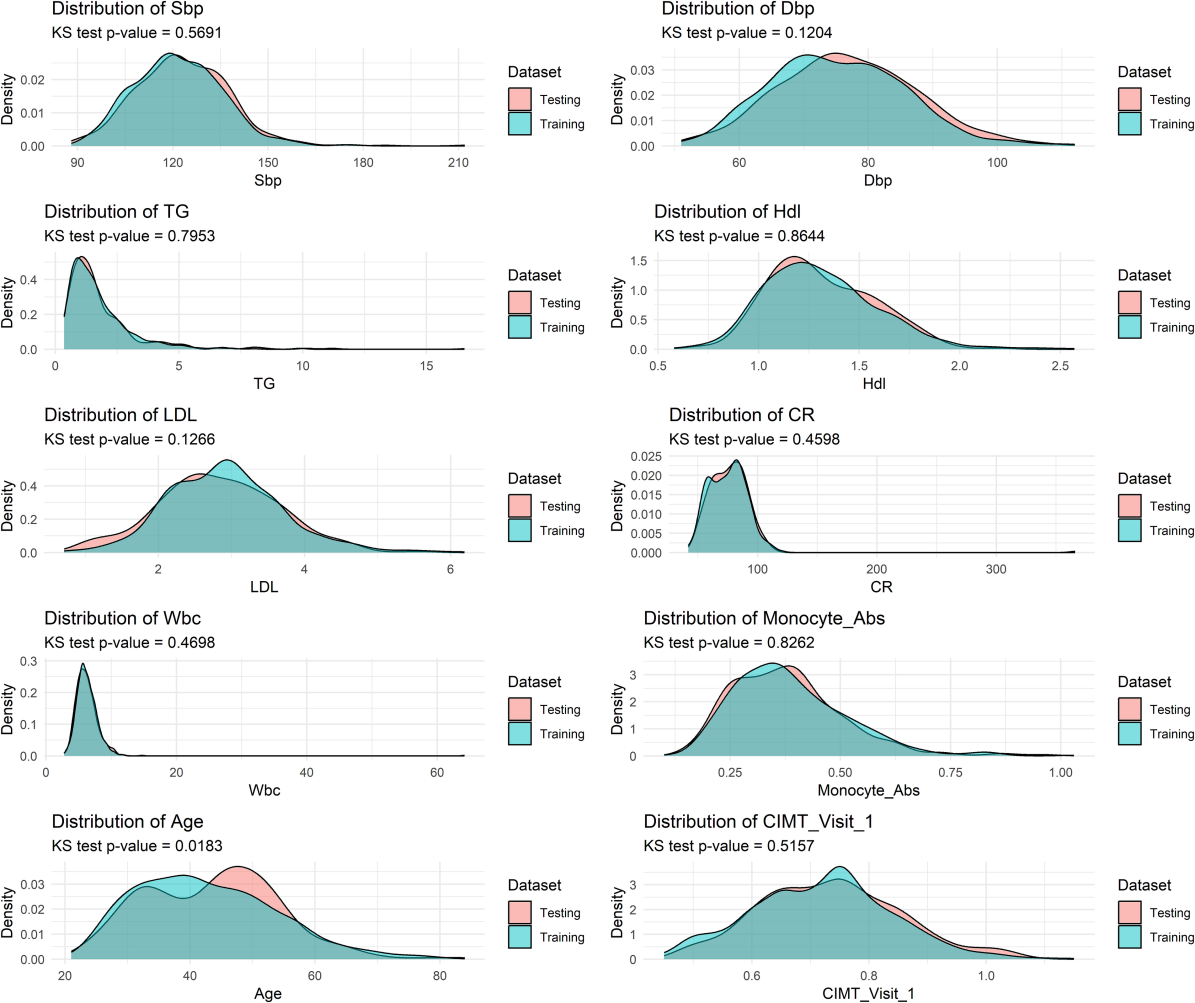
Kolmogorov–Smirnov tests for training and testing sets.

To evaluate potential covariate shifts between the training and test datasets, we conducted Kolmogorov–Smirnov tests for all the input features (Figure 3). The results revealed that eight out of nine features presented no significant distributional differences between the training and test datasets. Only age demonstrated a statistically significant distributional discrepancy (*p* = 0.0183).

Notably, despite this age distribution difference, our model maintained robust performance in the test set (AUC >0.7), indicating a degree of resilience to age-related covariate shifts. This result strengthens our confidence in the model’s generalizability, suggesting that it may maintain stable predictive performance when confronted with minor population distribution differences in real-world scenarios.

### 3.4 Model performance comparison

**Figure 4:**
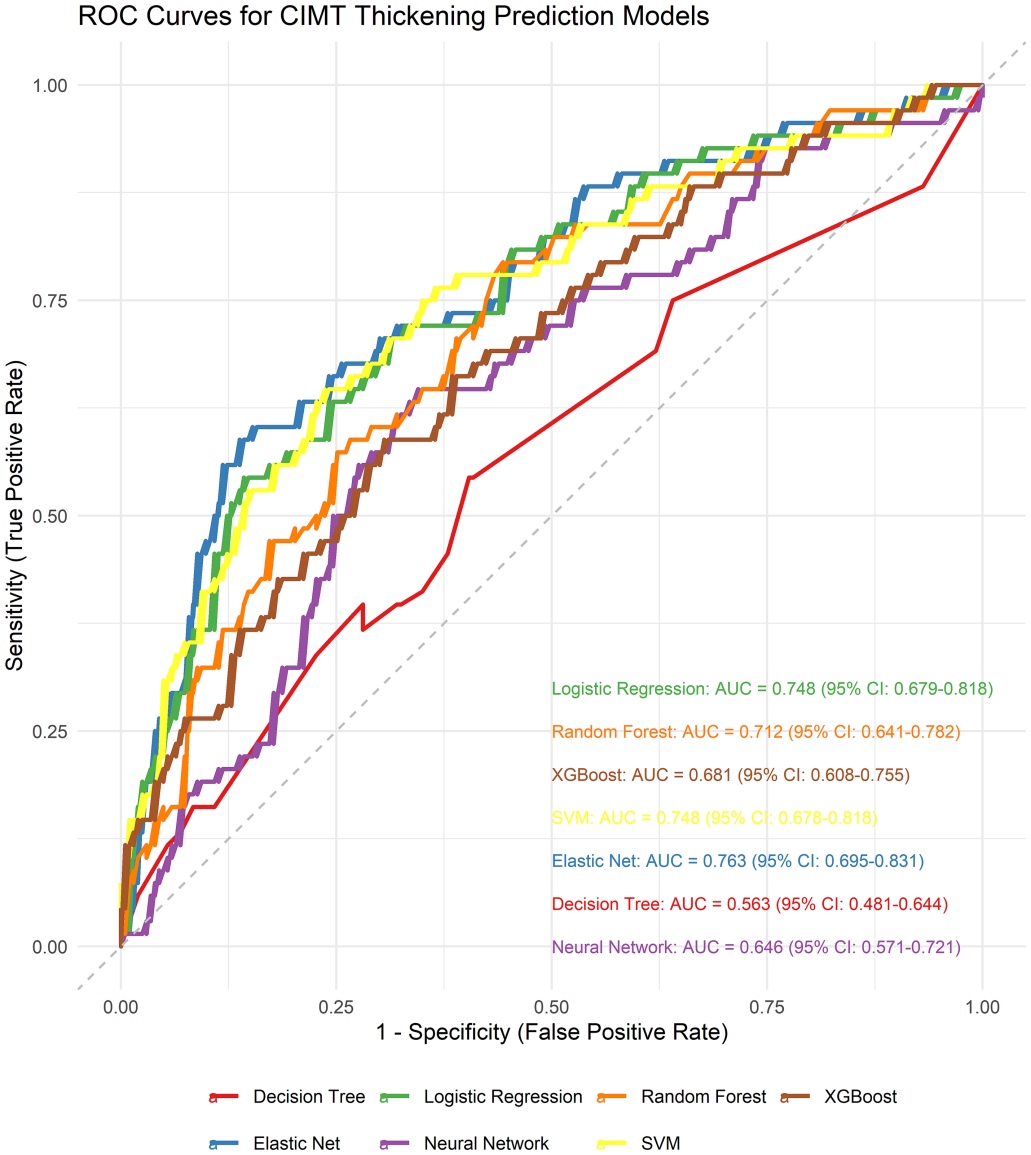
Receiver operating characteristic (ROC) curves comparing the discriminative performance of seven machine learning models.

We generated seven ML algorithms to predict patient CIMT thickening within three years. Figure 4 and Table 1 show the discriminative performance of the nine models in terms of their ROC curves.

**Table 1:** Performance comparison of seven machine learning models for predicting CIMT thickening.

Model	AUC (lower CI–upper CI)	Accuracy (lower CI–upper CI)	Sensitivity (lower CI–upper CI)	Specificity (lower CI–upper CI)	PPV (lower CI–upper CI)	NPV (lower CI–upper CI)	*F*_1_ (lower CI–upper CI)	LogLoss (lower CI–upper CI)
Elastic Net	0.763 (0.695–0.831)	0.701 (0.646–0.753)	0.706 (0.603–0.814)	0.700 (0.638–0.763)	0.440 (0.345–0.535)	0.877 (0.827–0.923)	0.542 (0.446–0.622)	0.603 (0.576–0.636)
Logistic Regression	0.748 (0.679–0.818)	0.694 (0.631–0.745)	0.676 (0.558–0.790)	0.700 (0.627–0.758)	0.430 (0.330–0.525)	0.866 (0.815–0.914)	0.526 (0.432–0.617)	0.589 (0.539–0.652)
SVM	0.748 (0.678–0.818)	0.731 (0.679–0.779)	0.647 (0.523–0.750)	0.759 (0.699–0.814)	0.473 (0.375–0.568)	0.865 (0.815–0.912)	0.547 (0.446–0.631)	0.575 (0.501–0.657)
Random Forest	0.712 (0.641–0.782)	0.734 (0.675–0.786)	0.471 (0.356–0.590)	0.823 (0.760–0.871)	0.471 (0.352–0.587)	0.823 (0.769–0.873)	0.471 (0.364–0.563)	0.556 (0.516–0.602)
XGBoost	0.681 (0.608–0.755)	0.694 (0.633–0.749)	0.456 (0.344–0.574)	0.773 (0.708–0.833)	0.403 (0.289–0.515)	0.809 (0.756–0.865)	0.428 (0.315–0.526)	0.685 (0.583–0.802)
Neural Network	0.646 (0.571–0.721)	0.661 (0.605–0.720)	0.588 (0.469–0.701)	0.685 (0.619–0.746)	0.385 (0.290–0.473)	0.832 (0.769–0.886)	0.465 (0.359–0.555)	0.893 (0.745–1.056)
Decision Tree	0.563 (0.481–0.644)	0.579 (0.520–0.638)	0.456 (0.324–0.580)	0.621 (0.545–0.690)	0.287 (0.197–0.369)	0.773 (0.712–0.835)	0.352 (0.253–0.439)	2.309 (1.470–3.164)

**Figure 5:**
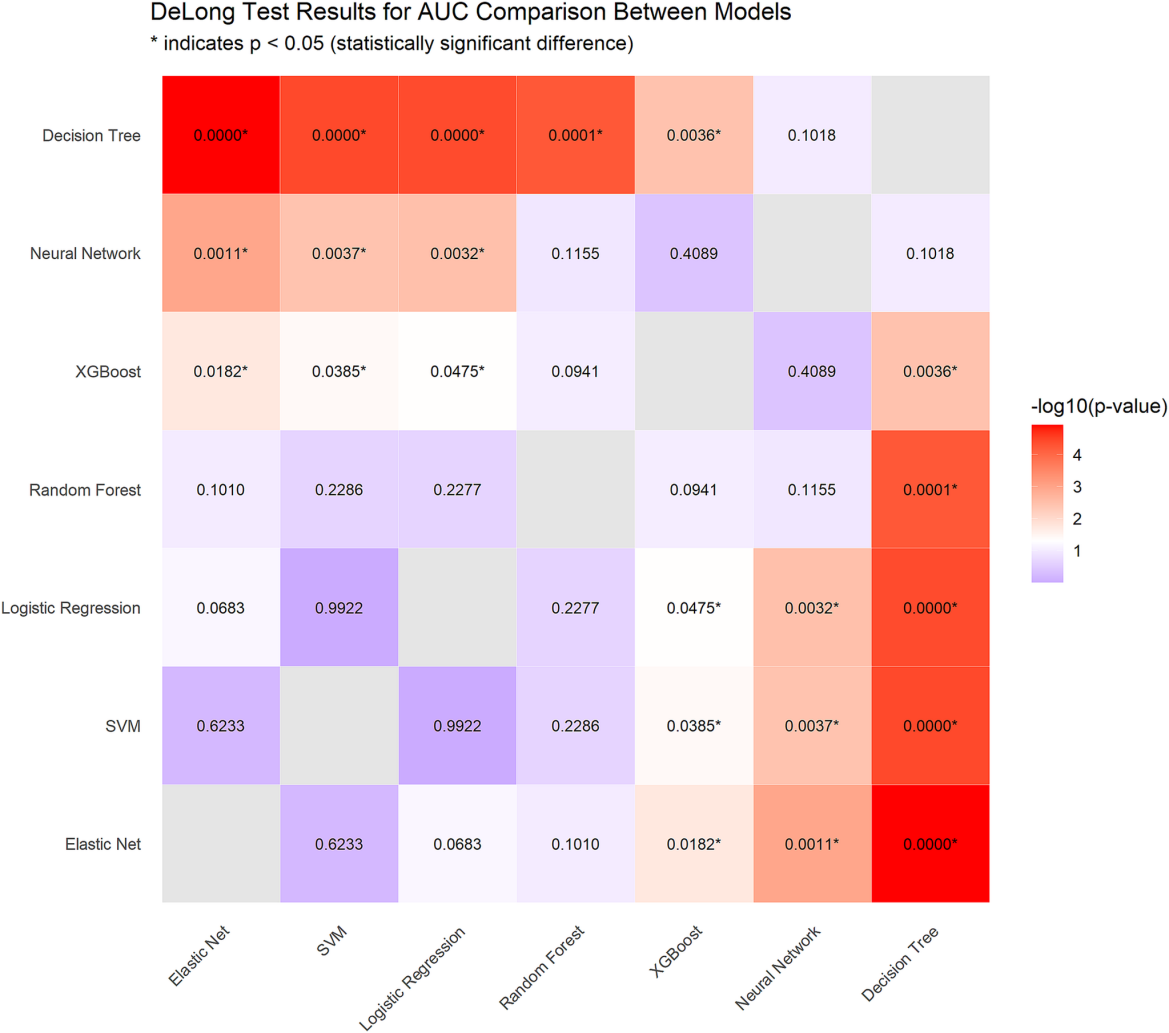
DeLong test results for AUC comparison between models.

To identify the optimal model, we performed DeLong tests (Figure 5). The results revealed no statistically significant differences in the AUC among the elastic net, logistic regression, and SVM methods (*p* = 0.623 and *p* = 0.992, respectively), suggesting the need for further comprehensive analysis.

Using paired bootstrap *t*-tests (1,000 resamples), we calculated the performance differences between the models. For the AUC, Elastic Net outperformed logistic regression by an average of 0.0140 (*p* < 0.001) and SVM by 0.0146 (*p* < 0.001). In terms of sensitivity, Elastic Net demonstrated superiority over logistic regression by 0.0294 (*p* < 0.001) and over SVM by 0.0579 (*p* < 0.001).

To comprehensively evaluate the three models, we implemented a multimetric weighted scoring approach, assigning weights to the AUC, sensitivity, *F*_1_-score, and log loss according to clinical relevance (30, 30, 20, and 20%, respectively). Elastic Net achieved the highest score (0.628), followed by logistic regression (0.615) and SVM (0.613).

Considering statistical significance testing, weighted scoring results, and Elastic Net’s intrinsic feature selection capabilities, we selected Elastic Net as the optimal model for subsequent analyses.

**Figure 6:**
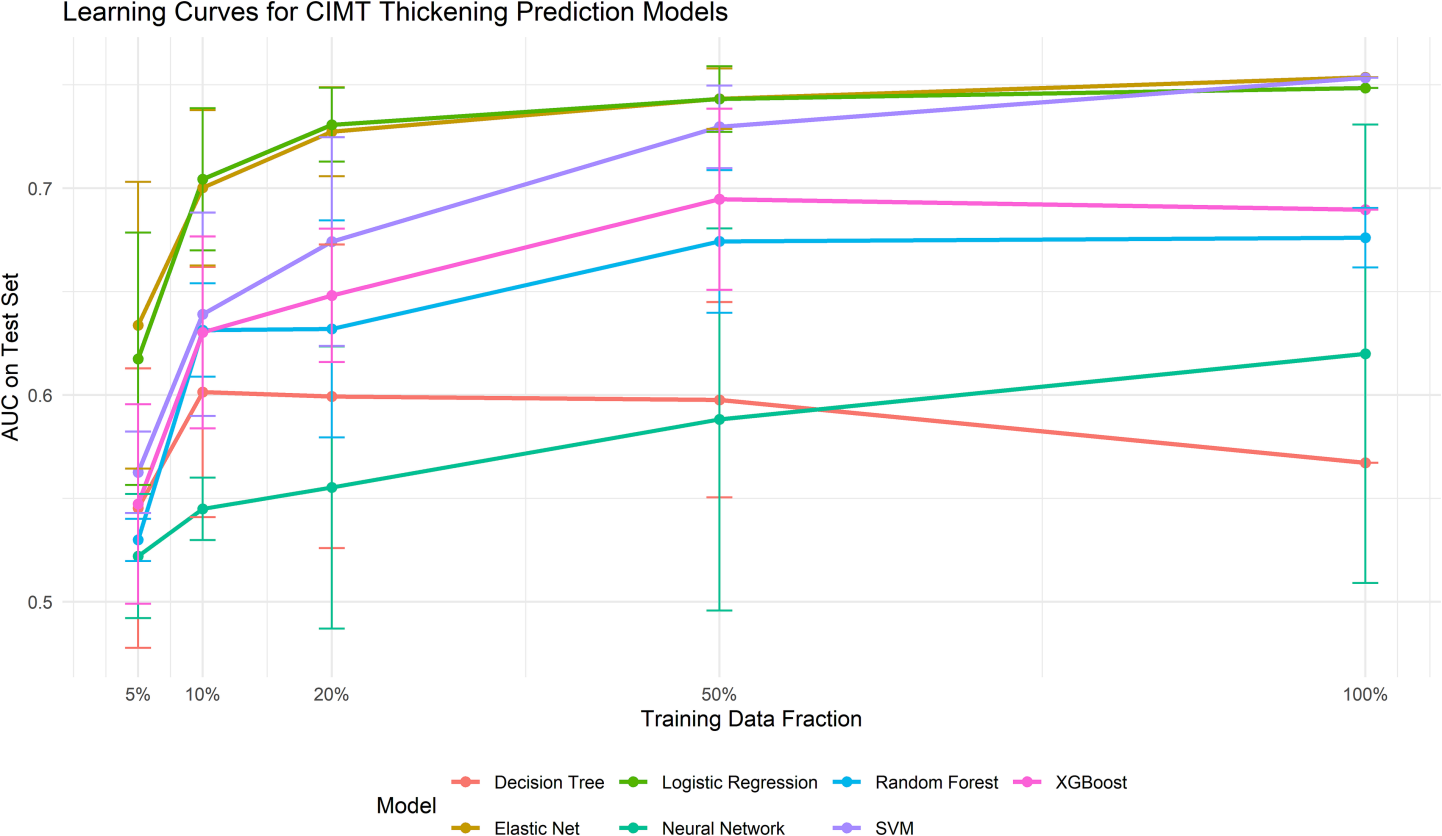
Learning curves for each model.

To address potential overfitting and underfitting concerns, we constructed learning curves (Figure 6). Analysis revealed that Elastic Net consistently demonstrated superior performance across all training data volumes (AUC improvement from 0.634 with 5% data to 0.754 with 100% data). Notably, the three top-performing models—Elastic Net, logistic regression, and SVM—achieved AUCs exceeding 0.70 even with minimal data (10%), indicating excellent data efficiency. This analysis further validated Elastic Net’s stability and superiority while confirming that the current data volume was sufficient for model training (Table 2).

**Table 2:** Learning curve performance of machine learning models with varying training data volumes.

Model	5% training data (32 samples)	10% training data (63 samples)	20% training data (127 samples)	50% training data (316 samples)	100% training data (633 samples)
Elastic Net	0.634 ± 0.069	0.700 ± 0.038	0.727 ± 0.022	0.743 ± 0.015	0.754 ± 0.000
SVM	0.563 ± 0.020	0.639 ± 0.049	0.674 ± 0.050	0.730 ± 0.020	0.753 ± 0.000
Logistic Regression	0.617 ± 0.061	0.704 ± 0.034	0.731 ± 0.018	0.743 ± 0.016	0.748 ± 0.000
XGBoost	0.547 ± 0.048	0.630 ± 0.046	0.648 ± 0.032	0.695 ± 0.044	0.690 ± 0.000
Random Forest	0.530 ± 0.010	0.631 ± 0.023	0.632 ± 0.052	0.674 ± 0.034	0.676 ± 0.014
Neural Network	0.522 ± 0.030	0.545 ± 0.015	0.555 ± 0.068	0.588 ± 0.092	0.620 ± 0.111
Decision Tree	0.545 ± 0.068	0.601 ± 0.061	0.599 ± 0.073	0.598 ± 0.047	0.567 ± 0.000

Model performance on test set with varying training data sizes. Test set performance measured by AUC (area under ROC curve).

### 3.5 Model calibration performance

**Figure 7:**
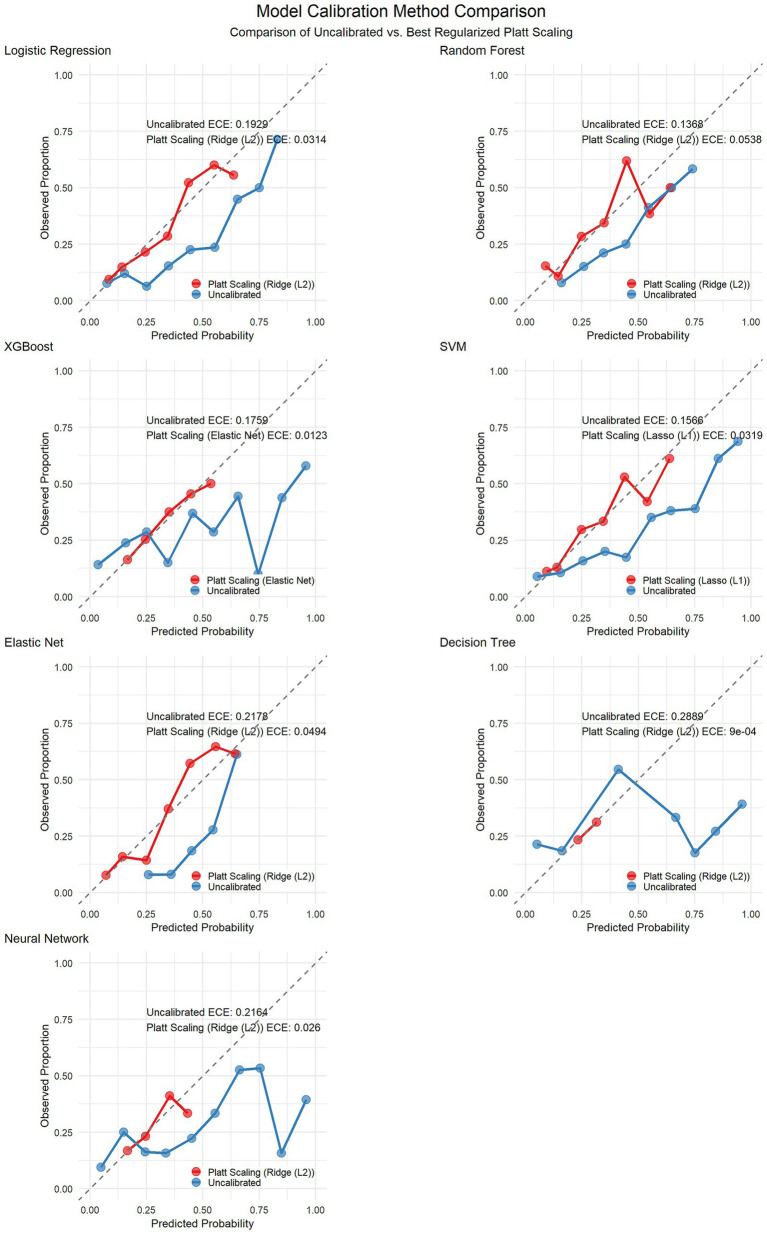
Calibration curves before and after Platt scaling correction for seven machine learning models.

To increase the reliability of the predictive probabilities, we implemented Platt scaling across all the models via three regularization methods (ridge L2, lasso L1, and elastic net) combined with stratified k-fold cross-validation to prevent overfitting. The optimal calibration methods varied by model: both elastic net and logistic regression performed best with ridge regularization, whereas SVM yielded superior results with lasso regularization, highlighting the influence of model characteristics on the selection of the calibration method. Calibration not only improved the ECE but also significantly enhanced metrics such as the Brier score and log loss (see Table 3). Figure 7 shows the calibration curves of each model before and after Platt scaling.

**Table 3:** Calibration performance metrics of the models before and after Platt scaling.

Model	Best calibration method	ECE (before calibration)	ECE (after calibration)	ECE improvement rate (%)	Brier score (before calibration)	Brier score (after calibration)	Brier score improvement rate (%)	LogLoss (before calibration)	LogLoss (after calibration)	LogLoss improvement rate (%)
Logistic Regression	Ridge (L2)	0.1929	0.0314	83.7	0.2008	0.16	20.3	0.5891	0.4921	16.5
Random Forest	Ridge (L2)	0.1368	0.0538	60.7	0.1874	0.1709	8.8	0.5564	0.5172	7
XGBoost	Elastic Net	0.1759	0.0123	93	0.2166	0.1771	18.2	0.6847	0.5353	21.8
SVM	Lasso (L1)	0.1566	0.0319	79.6	0.191	0.1587	16.9	0.575	0.4888	15
Elastic Net	Ridge (L2)	0.2178	0.0494	77.3	0.2069	0.1556	24.8	0.6031	0.4819	20.1
Decision Tree	Ridge (L2)	0.2889	0.0009	99.7	0.3148	0.1864	40.8	2.3094	0.5594	75.8
Neural Network	Ridge (L2)	0.2164	0.026	88	0.2567	0.1805	29.7	0.8926	0.5425	39.2

### 3.6 Feature importance analysis

**Figure 8:**
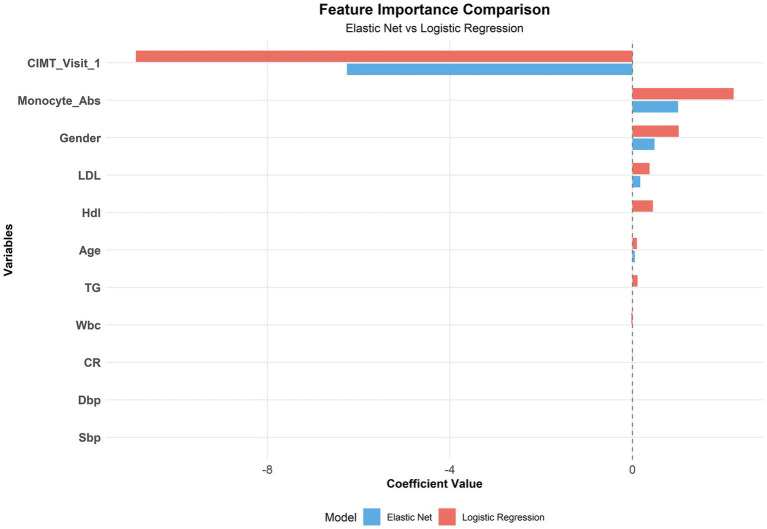
Comparison of the feature importance between the elastic net and logistic regression.

Although Elastic Net was identified as the optimal model, we conducted a comparative feature importance analysis between Elastic Net and logistic regression (both linear models) to provide more comprehensive feature selection insights. Both models identified the following variables as important predictors: baseline CIMT, absolute monocyte count, sex, age, and LDL-C (Figure 8).

### 3.7 Subgroup performance analysis

To assess model performance consistency across different patient populations, we conducted stratified analyses of the test set by age (≤35 years, 35–50 years, >50 years), sex (male, female), and baseline CIMT level (low: <0.6 mm, medium: 0.6–0.8 mm, high: >0.8 mm). The sample size distribution across subgroups is presented in Table 4.

**Table 4:** Predictive performance of the elastic net model across different subgroups.

Subgroup	Model	*N*	AUC	Sensitivity	Specificity
Age group = 35–50	Decision Tree	124	0.592	0.436	0.694
	Elastic Net		0.821	0.846	0.729
	Logistic Regression		0.812	0.769	0.718
	Neural Network		0.69	0.641	0.729
	Random Forest		0.761	0.59	0.918
	SVM		0.805	0.769	0.776
	XGBoost		0.744	0.513	0.894
Age group = >50	Decision Tree	71	0.51	0.375	0.473
	Elastic Net		0.598	0.5	0.582
	Logistic Regression		0.583	0.562	0.509
	Neural Network		0.615	0.5	0.655
	Random Forest		0.574	0.25	0.709
	SVM		0.637	0.375	0.764
	XGBoost		0.547	0.375	0.636
Age group = ≤35	Decision Tree	76	0.633	0.615	0.651
	Elastic Net		0.779	0.538	0.762
	Logistic Regression		0.761	0.538	0.841
	Neural Network		0.57	0.538	0.651
	Random Forest		0.712	0.385	0.794
	SVM		0.731	0.615	0.73
	XGBoost		0.669	0.385	0.73
CIMT group = High	Decision Tree	65	0.479	0.3	0.673
	Elastic Net		0.484	0.1	0.855
	Logistic Regression		0.529	0.1	0.782
	Neural Network		0.502	0.1	0.745
	Random Forest		0.577	0.1	0.818
	SVM		0.538	0.2	0.818
	XGBoost		0.609	0.1	0.745
CIMT group = Low	Decision Tree	49	0.629	0.611	0.613
	Elastic Net		0.801	1	0.419
	Logistic Regression		0.79	0.944	0.516
	Neural Network		0.642	0.833	0.419
	Random Forest		0.741	0.611	0.742
	SVM		0.767	0.889	0.516
	XGBoost		0.647	0.5	0.581
CIMT group = Medium	Decision Tree	157	0.535	0.425	0.598
	Elastic Net		0.786	0.725	0.701
	Logistic Regression		0.775	0.7	0.709
	Neural Network		0.654	0.6	0.726
	Random Forest		0.754	0.5	0.846
	SVM		0.776	0.65	0.795
	XGBoost		0.755	0.525	0.838
Sex = Female	Decision Tree	98	0.606	0.591	0.632
	Elastic Net		0.827	0.727	0.803
	Logistic Regression		0.824	0.682	0.816
	Neural Network		0.755	0.727	0.75
	Random Forest		0.812	0.409	0.882
	SVM		0.782	0.682	0.789
	XGBoost		0.737	0.409	0.816
Sex = Male	Decision Tree	173	0.541	0.391	0.614
	Elastic Net		0.73	0.696	0.638
	Logistic Regression		0.712	0.674	0.63
	Neural Network		0.589	0.522	0.646
	Random Forest		0.662	0.5	0.787
	SVM		0.726	0.63	0.74
	XGBoost		0.648	0.478	0.748

**Figure 9:**
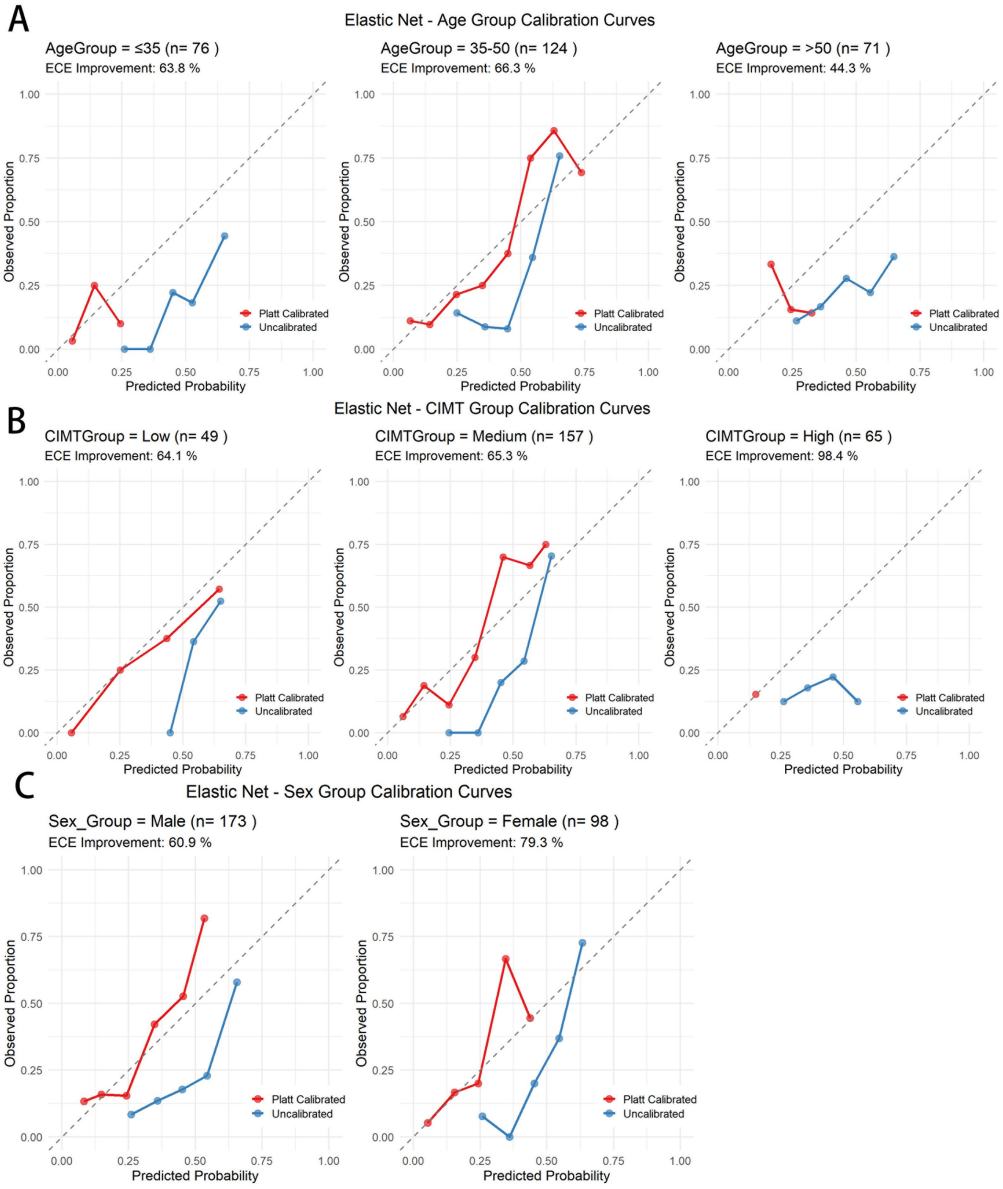
Calibration curves before and after Platt scaling for different subgroups in the elastic net model. **(A)** Age subgroups. **(B)** CIMT thickness subgroups. **(C)** Gender subgroups.

To ensure calibration performance across subgroups, we applied Platt scaling to the elastic net model (Table 5). All the subgroups demonstrated improvement. Figure 9 presents the ECE improvement before and after calibration. The predictive performance for the older age and high baseline CIMT subgroups was significantly lower than that for the other groups, suggesting increased prediction difficulty in these populations. Compared with male subjects, female subjects consistently demonstrated superior prediction performance, indicating sex-related prediction bias that warrants consideration in clinical applications. Despite varying initial calibration levels across subgroups, Platt scaling achieved substantial calibration improvements in all subgroups, confirming the robustness of the calibration methodology (see Table 6).

**Table 5:** Calibration performance improvement of the elastic net model before and after Platt scaling across different subgroups.

Model	Group	Subgroup	*N*	ECE_uncalibrated	ECE_calibrated	Improvement
Elastic Net	Age	35–50	124	0.2224	0.0749	66.3
		≤35	76	0.2719	0.0984	63.8
		>50	71	0.2515	0.1401	44.3
	CIMT	High	65	0.2128	0.0035	98.4
		Low	49	0.2061	0.0739	64.1
		Medium	157	0.2433	0.0844	65.3
	Sex	Female	98	0.2345	0.0485	79.3
		Male	173	0.2233	0.0872	60.9

**Table 6:** Comparison of optimal decision thresholds and discriminative metrics before and after Platt scaling across machine learning models.

Model	Calibration	Best threshold	Youden index	Sensitivity	Specificity
Elastic Net	Original	0.57	0.45	0.588	0.862
	Calibrated	0.36	0.445	0.588	0.857
SVM	Original	0.53	0.406	0.632	0.773
	Calibrated	0.3	0.406	0.632	0.773
Logistic Regression	Original	0.62	0.391	0.544	0.847
	Calibrated	0.37	0.391	0.544	0.847
Random Forest	Original	0.36	0.336	0.779	0.557
	Calibrated	0.2	0.346	0.794	0.552
Neural Network	Original	0.48	0.297	0.618	0.68
	Calibrated	0.26	0.283	0.603	0.68
XGBoost	Original	0.36	0.283	0.588	0.695
	Calibrated	0.25	0.283	0.588	0.695
Decision Tree	Original	0.17	0.14	0.544	0.596
	Calibrated	0.23	0.14	0.544	0.596

### 3.8 Decision curve analysis

To evaluate the clinical utility of the Elastic Net model for predicting CIMT thickening, we conducted decision curve analysis (DCA) and Platt calibration-based risk stratification. See Supplementary material 2 for the DCA graphs of each model.

**Figure 10:**
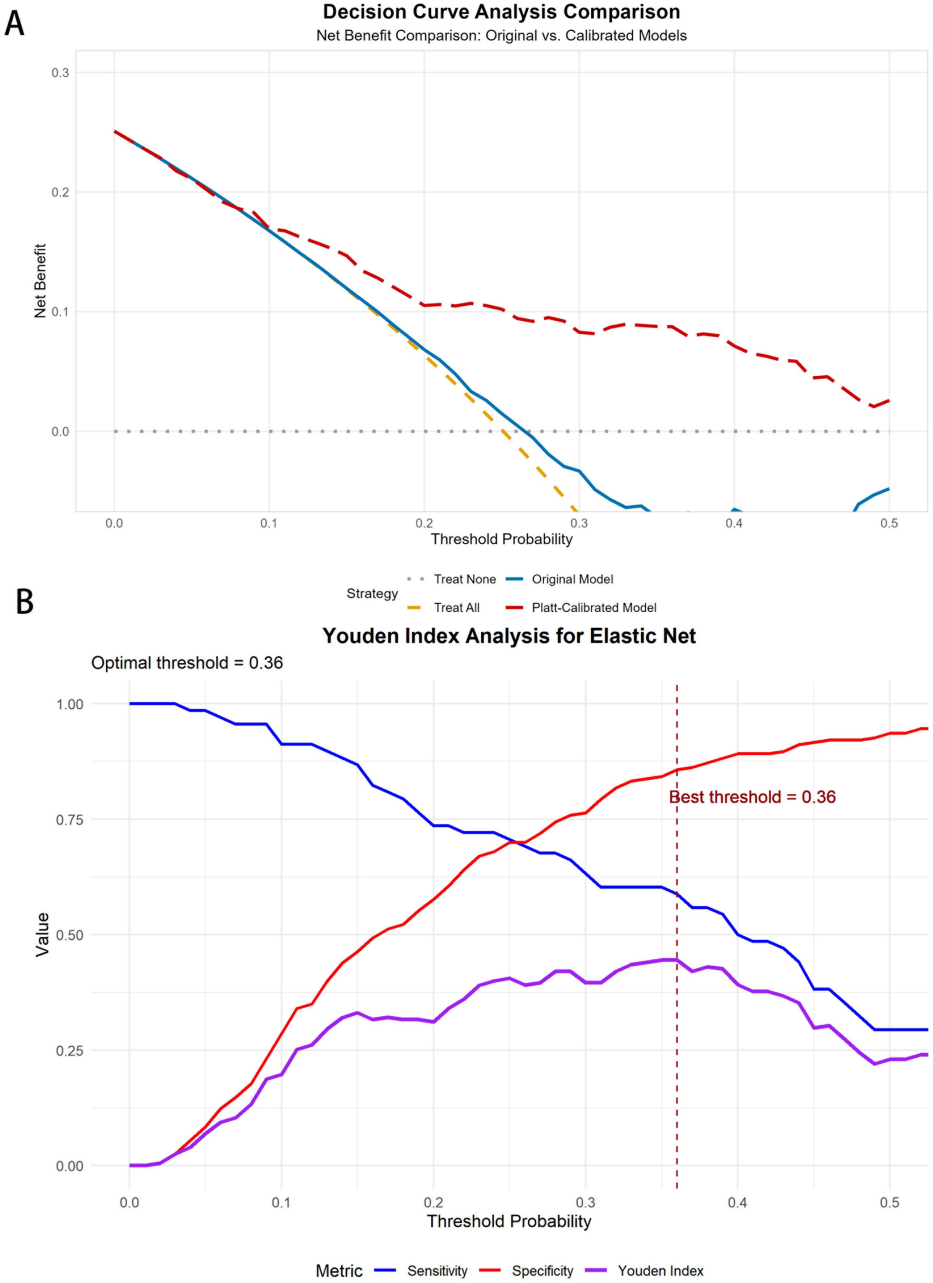
DCA curves and Youden curves for the elastic net. **(A)** DCA curves for elastic net before and after Platt scaling. **(B)** Youden index analysis for elastic net.

Youden index analysis revealed that the optimal threshold decreased from 0.57 (sensitivity 0.588, specificity 0.862) in the original model to 0.36 (sensitivity 0.588, specificity 0.857) after calibration, while maintaining similar discriminative ability (Youden index ≈0.45) but providing more accurate probability estimates. The DCA revealed a maximum net benefit threshold of 0.01 (net benefit value 0.243), with positive net benefit maintained across the threshold range of 0.01–0.5, demonstrating the model’s clinical utility across a broad range of thresholds (Figure 10).

On the basis of calibrated probabilities and clinical risk stratification, patients were classified into three groups: a medium-risk group (probability <0.36), comprising 202 individuals with an event rate of 13.9%; a high-risk group (probability 0.36–0.41), comprising 14 individuals with an event rate of 50.0%; and a very-high-risk group (probability ≥0.41), comprising 55 individuals with an event rate of 60.0%. This stratification demonstrated a clear risk gradient, providing an objective basis for clinical intervention.

On the basis of these risk stratification results, we recommend differentiated intervention strategies: for the medium-risk group (13.9% event rate), regular follow-up and lifestyle guidance; for the high-risk group (50% event rate), intensified lifestyle interventions and consideration of pharmacological therapy; and for the very-high-risk group (60% event rate), aggressive pharmacological intervention and close monitoring. This stratified intervention approach facilitates the optimization of healthcare resource allocation and enhances cost-effectiveness in preventing and managing CIMT.

## 4 Discussion

In this three-year prospective cohort study, we developed and validated machine learning models based on routine clinical biomarkers for predicting CIMT progression. Our findings demonstrate that machine learning approaches, particularly the elastic net model, can effectively identify individuals at high risk for CIMT thickening, thereby supporting targeted preventive interventions for atherosclerosis.

Our comprehensive evaluation of seven diverse machine learning algorithms was strategically designed to cover different modeling paradigms. The selection of these specific algorithms was based on several considerations: (1) linear models (logistic regression, elastic net) for interpretability and regularization capabilities; (2) tree-based models (decision tree, random forest, XGBoost) for their ability to capture nonlinear relationships and interactions without requiring extensive feature engineering; (3) kernel-based methods (SVMs) for their effectiveness with high-dimensional data and complex decision boundaries; and (4) neural networks for their potential to model complex patterns through multiple layers of abstraction. This diverse algorithmic approach allowed us to assess whether linear or nonlinear methods were better suited for CIMT progression prediction.

Interestingly, our comparative analysis revealed that simpler models (elastic net, LR, and SVM) outperformed complex algorithms such as random forest and neural networks in our dataset. This finding aligns with previous research indicating that when sample sizes are moderate (as in our study with *n* = 904) and relationships between predictors and outcomes are predominantly linear, simpler models often perform better than or at least comparably to complex models (27). Additionally, these models have a lower risk of overfitting, which is crucial for ensuring generalizability in clinical applications. The superior performance of the elastic net suggests that the relationship between clinical biomarkers and CIMT progression may be more linear than complex interactions.

A key strength of our study was the implementation of Platt scaling for probability calibration. Our analysis demonstrated that the original models, despite having good discrimination (AUC), produced probability estimates that were not well calibrated, particularly for the neural network and decision tree algorithms, which presented high expected calibration error (ECE) values. By applying Platt scaling with appropriate regularization methods (ridge for elastic net and logistic regression, lasso for SVM), we significantly improved the calibration performance across all the models, with the most dramatic improvements observed in the more complex models.

The significant improvement in the calibration metrics has profound clinical implications. Well-calibrated models provide reliable probability estimates that directly correspond to observed event rates, which is essential for accurate risk stratification in clinical practice (28). When physicians rely on predicted probabilities to guide treatment decisions, poorly calibrated models may lead to inappropriate interventions or missed prevention opportunities (29). Our findings emphasize that when developing clinical prediction tools, attention should be given not only to discrimination metrics such as the AUC but also to ensuring good calibration performance.

Our decision curve analysis (DCA) further validated the clinical utility of the calibrated elastic net model, which demonstrated positive net benefit across a wide range of threshold probabilities (0.01–0.5). The DCA revealed that our model outperformed both the “treat all” and “treat none” strategies within this threshold range, indicating that selective intervention on the basis of our model’s predictions would provide better clinical outcomes than would treating either everyone or no one. The maximum net benefit was observed at a threshold of 0.01 (net benefit value 0.243), suggesting high utility even at low-risk thresholds, while maintaining positive net benefit up to a threshold of 0.5 demonstrated robust clinical applicability across diverse decision-making preferences.

On the basis of our calibrated probability estimates and decision curve analysis, we developed a three-tier risk stratification framework that identified distinct groups with progressively higher event rates: medium-risk (13.9%), high-risk (50.0%), and very-high-risk (60.0%) groups. Youden index analysis revealed that the optimal threshold decreased from 0.57 in the original model to 0.36 after calibration while maintaining similar discriminative ability (Youden index ≈0.45) but providing more accurate probability estimates. This finding underscores the importance of proper calibration for clinical threshold determination.

We combined the absolute threshold cutoff (baseline CIMT ≥1.0 mm) with a dynamic progression warning (increase ≥0.1 mm during follow-up), which, compared with traditional single-dimensional criteria, can both identify structural lesions (baseline values indicating irreversible arterial wall remodeling) and capture active progression (significant increases reflecting accelerated atherosclerotic processes, even when baseline values do not reach the threshold). This integrated criterion better aligns with the ‘cumulative-trigger’ two-stage model of cardiovascular events (30, 31).

CIMT values ≥1.0 mm, as a criterion for thickening, have been recognized in multiple international studies and guidelines (3) and are widely accepted as indicators of subclinical atherosclerosis. An increase of ≥0.1 mm in consecutive measurements reflects progressive changes in arterial wall structure, potentially indicating active progression of vascular lesions even when the absolute value has not reached the 1.0 mm threshold. Multiple prospective studies have shown that rapid CIMT progression is associated with increased cardiovascular event risk. Moreover, evidence suggests that CIMT progression itself (independent of baseline values) is associated with increased cardiovascular event risk (32, 33).

Moreover, the finding that the baseline CIMT is the strongest predictor aligns with previous research suggesting that subclinical atherosclerosis may promote further plaque development through mechanical and inflammatory mechanisms (34). The important contribution of inflammatory markers (monocyte count) in our model supports the increasingly recognized view that inflammation is a key driver of atherosclerotic progression (35–37).

Several limitations of our study warrant consideration. First and foremost, our model was developed and validated with data from a single center (Third Xiangya Hospital of Central South University Health Examination Cohort), which may limit its generalizability. The lack of external validation in diverse populations across different ethnic backgrounds, geographic regions, and healthcare settings represents a significant limitation that may lead to overestimation of the model’s actual applicability and performance in real-world settings. External validation across multiple diverse cohorts should be a priority for future research to establish the model’s true clinical value.

Second, while our comprehensive algorithm selection covered major machine learning paradigms, emerging deep learning approaches specifically designed for longitudinal data, such as recurrent neural networks or transformer models, were not evaluated. These methods might capture temporal patterns in CIMT progression more effectively and could be explored in future studies with larger datasets containing more temporal measurements.

Third, despite conducting subgroup analyses, the sample sizes for certain subgroups (particularly the >50 age group and high baseline CIMT group) were relatively small, which may have contributed to the observed performance limitations. The short and significantly deviating calibration curves in these subgroups reflect this limitation and suggest caution when applying the model to these populations. Future studies with enriched sampling of these challenging subgroups could help develop more robust prediction approaches for these specific populations.

Fourth, our feature set was limited to routinely available clinical and laboratory parameters. The incorporation of additional data modalities, such as genetic markers, advanced imaging features, or novel biomarkers of vascular inflammation, might increase the prediction accuracy, particularly for subgroups in which the current performance is suboptimal.

Finally, while our three-year follow-up period allows for meaningful assessment of CIMT progression, longer-term studies would provide valuable insights into the durability of prediction and the relationship between predicted CIMT progression and hard cardiovascular outcomes. The integration of cardiovascular event data strengthens the clinical relevance of our prediction model.

## Data Availability

The raw data supporting the conclusions of this article will be made available by the authors, without undue reservation.
