# Supplementary material for: Machine learning-based prediction of carotid intima–media thickness progression: a three-year prospective cohort study
Source: Front Med (Lausanne). 2025 Jun 12;12:1593662. doi: 10.3389/fmed.2025.1593662 (PMC12198118; doi:10.3389/fmed.2025.1593662)

**Figure 1:Decision Curve Analysis for Neural NetworkNet Benefit Comparison: Original vs. Calibrated Neural Network Miodel**


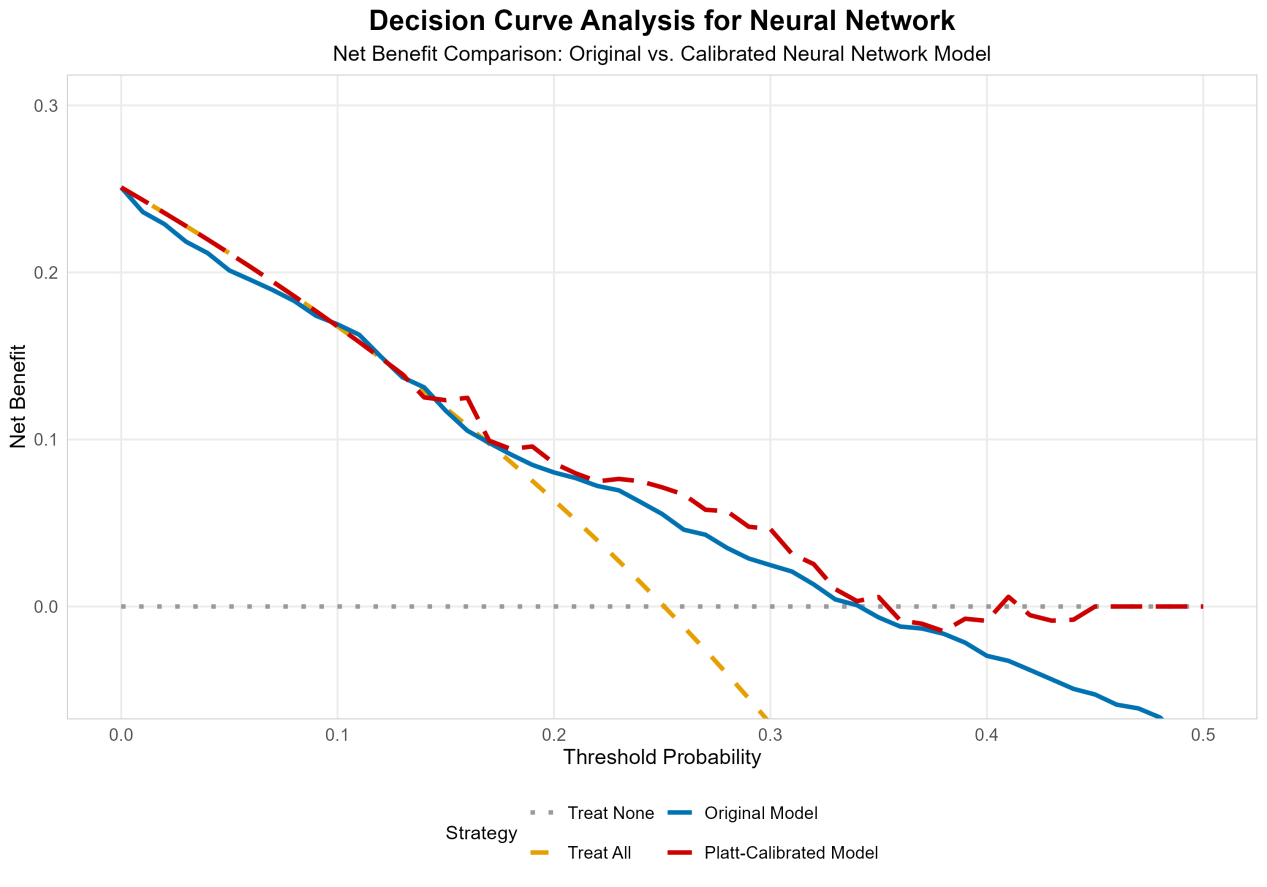


**Figure 2:Decision Curve Analysis for Random ForestNet Benefit Comparison: Original vs. Calibrated Random Forest Model**


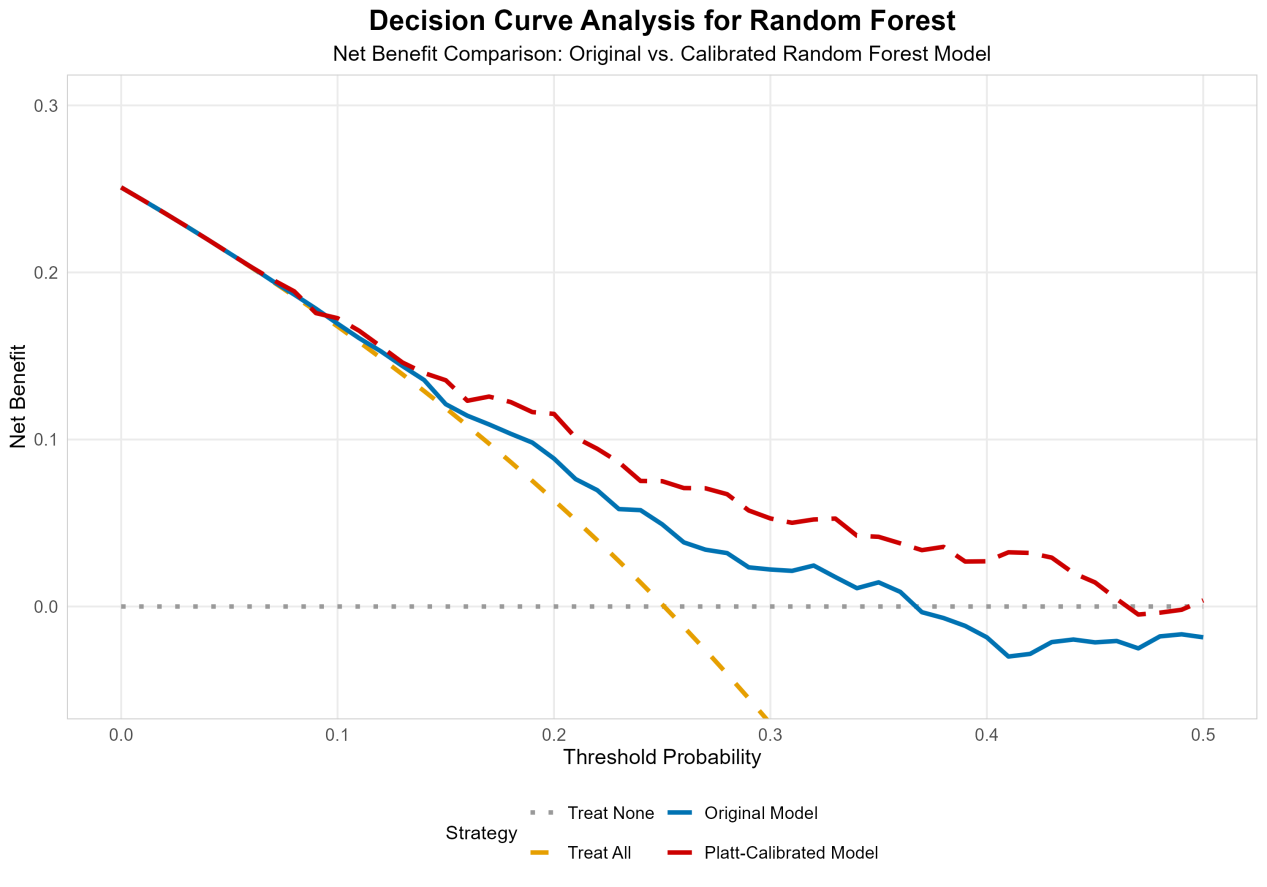


**Figure 3:Decision Curve Analysis for SVM Net Benefit Comparison: Original vs. Calibraled SVM Model**


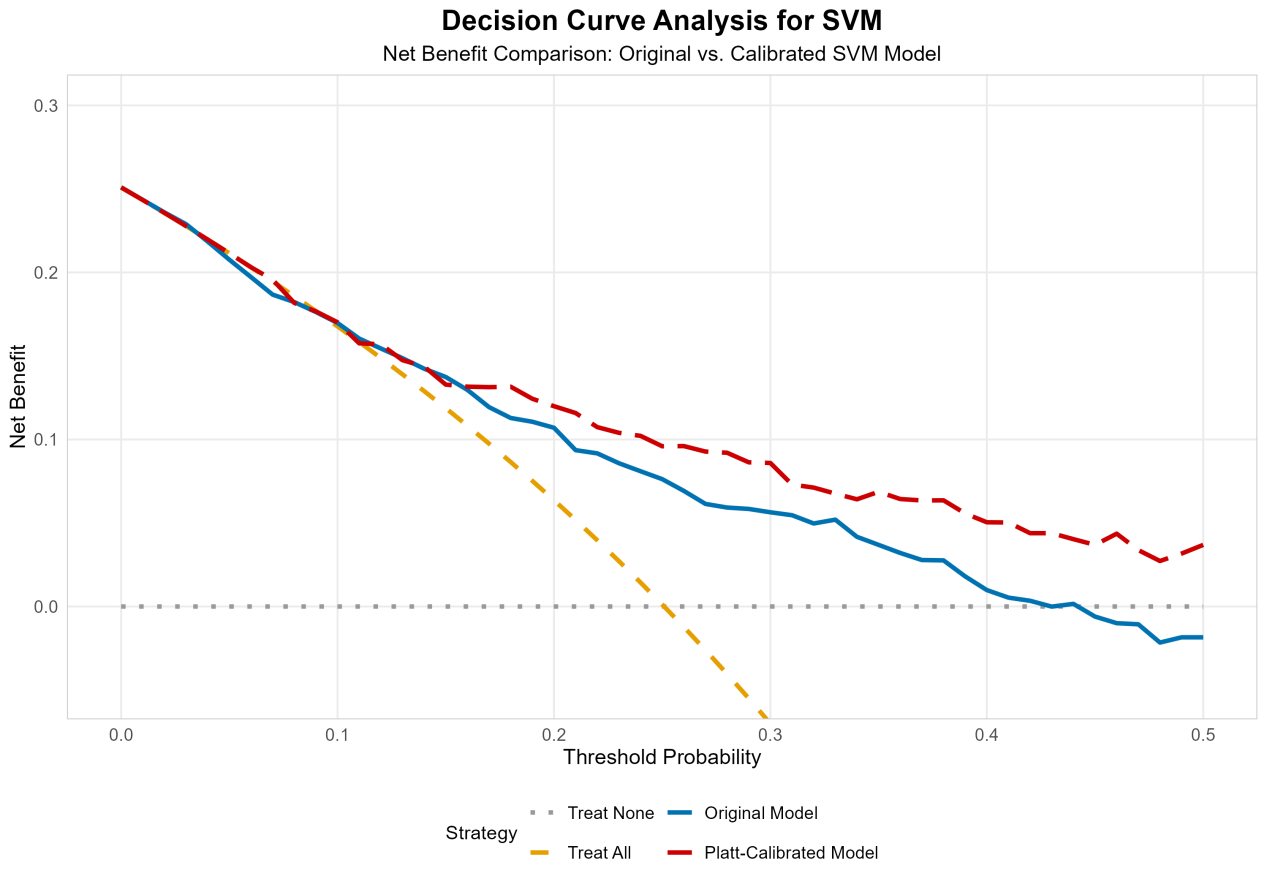


**Figure 4:Decision Curve Analysis for XGBoost Net Benefit Comparison: Original vs. Calibrated XGBoost Model**


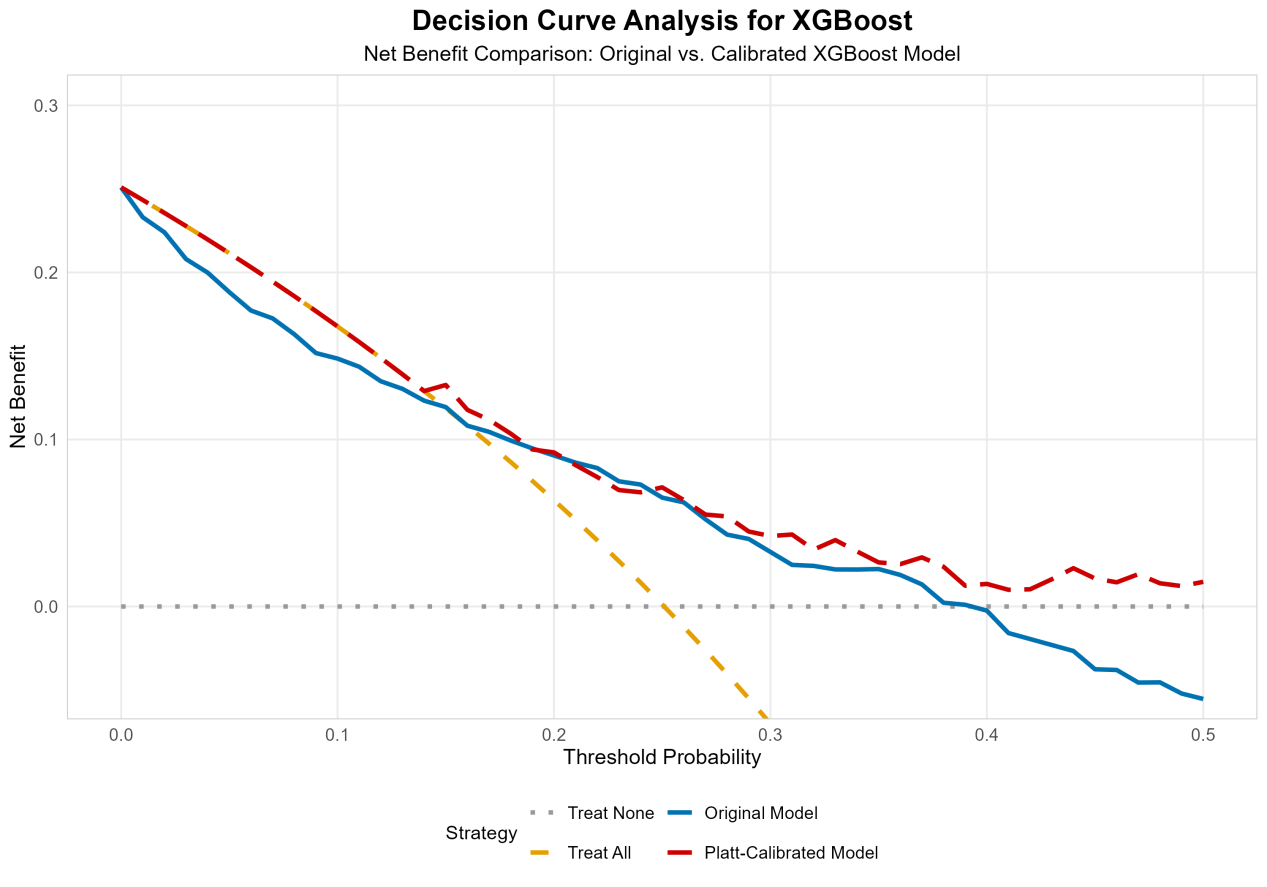


**Figure 5:Decision Curve Analysis for Decision Tree Net Beneft Comparison: Original vs. Calibrated Decision Tree Model**


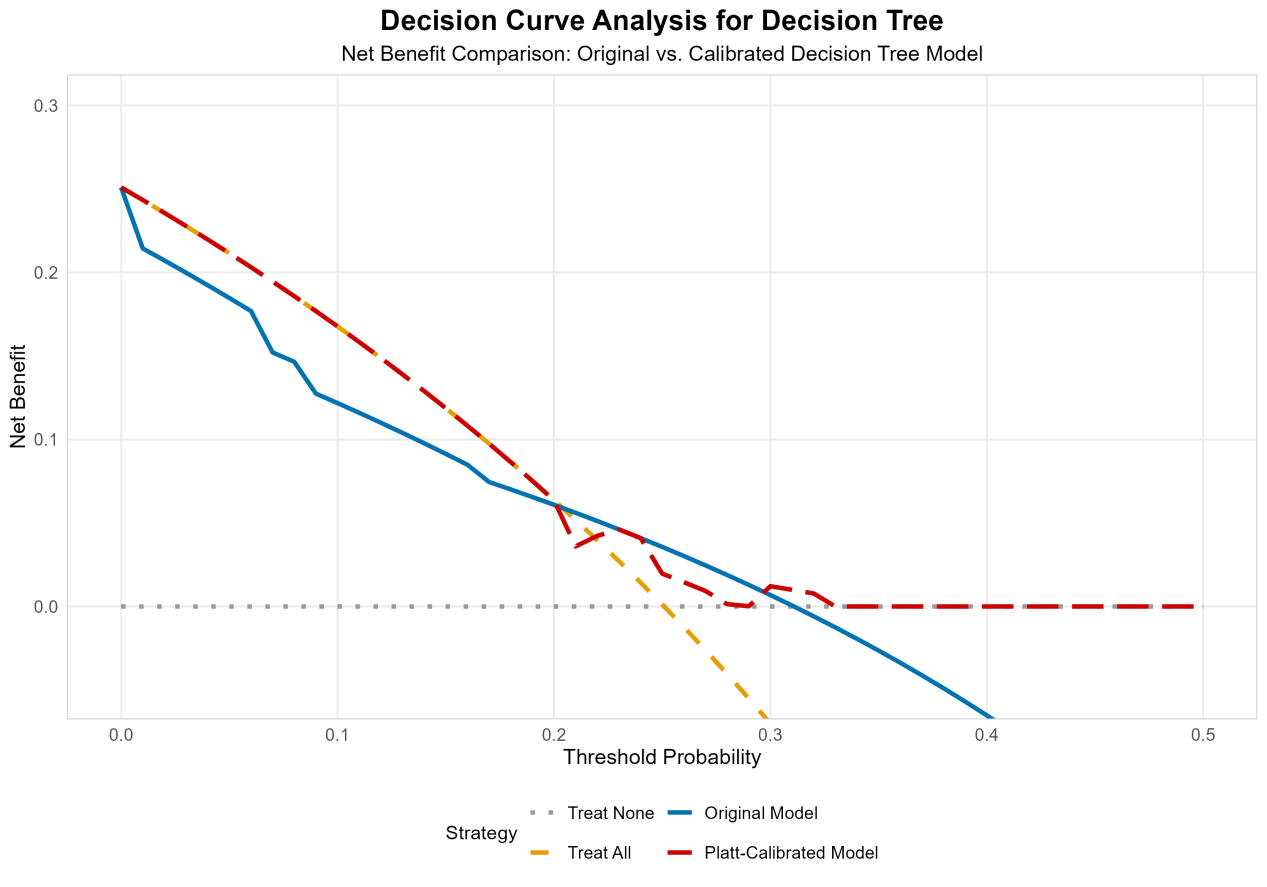
**Figure 6: Decision Curve Analysis for Logistic Regression Net Beneft Comparison: Original vs.Calibrated Logistic Regression Model**


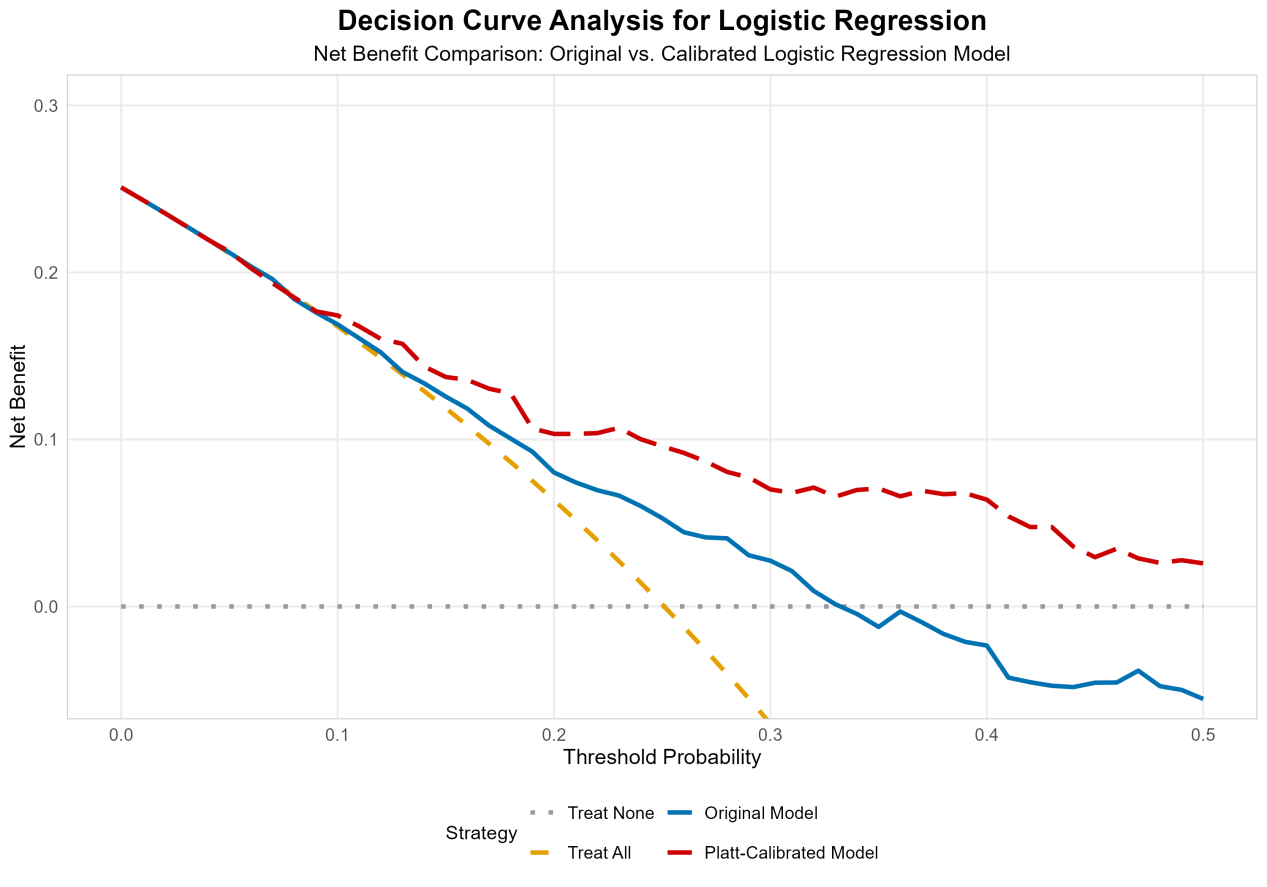

Supplement: Supplementary file 1 [file Data_Sheet_1.docx]
