# Supplementary material for: Machine learning-based prediction of carotid intima–media thickness progression: a three-year prospective cohort study
Source: Front Med (Lausanne). 2025 Jun 12;12:1593662. doi: 10.3389/fmed.2025.1593662 (PMC12198118; doi:10.3389/fmed.2025.1593662)
Supplement: Supplementary file 2 [file Table_1.docx]

| **Characteristic** | **Not Thickened** | **Thickened** | **p-value** | **SMD** |
| --- | --- | --- | --- | --- |
| n | 677 | 227 |  |  |
| MALE (%) | 427 (63.1) | 155 (68.3) | 0.181 | 0.110 |
| AGE (median [IQR]) | 42.00 [33.00, 51.00] | 43.00 [36.00, 50.00] | 0.119 | 0.125 |
| BMI (median [IQR]) | 24.20 [22.13, 26.22] | 24.12 [22.09, 25.85] | 0.568 | 0.049 |
| HEIGHT (MEDIAN [IQR]) | 165.60 [159.00, 170.50] | 166.50 [161.00, 171.05] | 0.120 | 0.137 |
| WEIGHT (MEDIAN [IQR]) | 66.00 [57.80, 75.10] | 66.00 [58.75, 75.50] | 0.640 | 0.029 |
| WAIST (MEDIAN [IQR]) | 83.00 [76.00, 90.00] | 83.00 [76.00, 89.00] | 0.933 | 0.005 |
| HIP (MEDIAN [IQR]) | 95.00 [91.00, 99.00] | 94.00 [91.00, 98.00] | 0.372 | 0.063 |
| SBP (MEDIAN [IQR]) | 122.00 [112.00, 132.00] | 121.00 [113.00, 131.00] | 0.919 | 0.017 |
| DBP (MEDIAN [IQR]) | 75.00 [68.00, 83.00] | 76.00 [68.25, 83.00] | 0.324 | 0.075 |
| PULSE (MEDIAN [IQR]) | 81.00 [74.00, 89.00] | 80.00 [75.00, 89.75] | 0.794 | 0.008 |
| TC (MEDIAN [IQR]) | 4.93 [4.32, 5.57] | 5.01 [4.38, 5.62] | 0.319 | 0.078 |
| TG (MEDIAN [IQR]) | 1.40 [0.89, 2.16] | 1.44 [0.94, 2.24] | 0.292 | 0.037 |
| HDL (MEDIAN [IQR]) | 1.26 [1.11, 1.48] | 1.29 [1.12, 1.48] | 0.625 | 0.014 |
| LDL (MEDIAN [IQR]) | 2.87 [2.31, 3.38] | 2.88 [2.40, 3.38] | 0.386 | 0.077 |
| HDL TC RATIO (MEDIAN [IQR]) | 0.26 [0.22, 0.31] | 0.26 [0.22, 0.31] | 0.688 | 0.046 |
| FBG (MEDIAN [IQR]) | 5.33 [5.01, 5.67] | 5.36 [5.08, 5.74] | 0.273 | 0.102 |
| ALT (MEDIAN [IQR]) | 22.00 [15.00, 34.00] | 21.00 [15.00, 31.00] | 0.766 | 0.029 |
| TBIL (MEDIAN [IQR]) | 12.80 [10.00, 16.20] | 12.50 [10.10, 16.25] | 0.949 | 0.046 |
| TP (MEDIAN [IQR]) | 75.10 [72.70, 78.00] | 75.95 [72.80, 78.80] | 0.053 | 0.137 |
| ALB (MEDIAN [IQR]) | 47.80 [45.90, 49.75] | 47.85 [46.20, 50.08] | 0.362 | 0.084 |
| GLOB (MEDIAN [IQR]) | 27.30 [25.10, 29.70] | 27.70 [25.42, 29.67] | 0.298 | 0.093 |
| BUN (MEDIAN [IQR]) | 4.56 [3.86, 5.38] | 4.55 [3.90, 5.44] | 0.534 | 0.056 |
| CR (MEDIAN [IQR]) | 76.00 [62.00, 86.00] | 77.00 [65.00, 86.00] | 0.425 | 0.097 |
| UA (MEDIAN [IQR]) | 353.00 [293.00, 413.50] | 359.00 [295.25, 423.00] | 0.417 | 0.067 |
| WBC (MEDIAN [IQR]) | 6.09 [5.22, 7.12] | 5.95 [5.20, 7.10] | 0.916 | 0.008 |
| RBC (MEDIAN [IQR]) | 4.93 [4.57, 5.26] | 4.94 [4.64, 5.24] | 0.849 | 0.028 |
| HGB (MEDIAN [IQR]) | 150.00 [137.00, 160.50] | 152.00 [139.00, 160.00] | 0.220 | 0.108 |
| HCT (MEDIAN [IQR]) | 45.20 [41.70, 47.85] | 45.60 [42.50, 47.90] | 0.311 | 0.075 |
| PLT (MEDIAN [IQR]) | 229.00 [196.00, 264.00] | 237.00 [204.00, 266.50] | 0.130 | 0.067 |
| NEUTROPHIL PCT (MEDIAN [IQR]) | 57.00 [51.85, 61.70] | 55.90 [49.90, 61.85] | 0.223 | 0.086 |
| LYMPHOCYTE PCT (MEDIAN [IQR]) | 33.90 [29.10, 38.90] | 34.90 [29.25, 40.15] | 0.464 | 0.053 |
| MONOCYTE PCT (MEDIAN [IQR]) | 6.00 [5.10, 7.00] | 6.20 [5.25, 7.10] | 0.151 | 0.103 |
| EOSINOPHIL PCT (MEDIAN [IQR]) | 1.90 [1.30, 3.00] | 2.10 [1.30, 3.30] | 0.450 | 0.057 |
| BASOPHIL PCT (MEDIAN [IQR]) | 0.30 [0.15, 0.60] | 0.30 [0.20, 0.60] | 0.930 | 0.028 |
| NEUTROPHIL ABS (MEDIAN [IQR]) | 3.41 [2.83, 4.15] | 3.31 [2.70, 4.23] | 0.581 | 0.007 |
| LYMPHOCYTE ABS (MEDIAN [IQR]) | 2.07 [1.69, 2.42] | 2.06 [1.73, 2.51] | 0.310 | 0.017 |
| MONOCYTE ABS (MEDIAN [IQR]) | 0.36 [0.28, 0.45] | 0.38 [0.30, 0.46] | 0.140 | 0.107 |
| EOSINOPHIL ABS (MEDIAN [IQR]) | 0.12 [0.08, 0.19] | 0.12 [0.07, 0.21] | 0.368 | 0.044 |
| BASOPHIL_ABS (MEDIAN [IQR]) | 0.02 [0.01, 0.03] | 0.02 [0.01, 0.03] | 0.840 | 0.015 |
| MCV (MEDIAN [IQR]) | 91.50 [88.85, 94.40] | 91.60 [89.50, 94.60] | 0.326 | 0.141 |
| MCH (MEDIAN [IQR]) | 30.40 [29.50, 31.40] | 30.60 [29.70, 31.40] | 0.171 | 0.187 |
| MCHC (MEDIAN [IQR]) | 331.00 [325.00, 337.00] | 332.00 [326.00, 338.00] | 0.072 | 0.163 |
| RDW (MEDIAN [IQR]) | 12.70 [12.40, 13.20] | 12.70 [12.40, 13.10] | 0.418 | 0.137 |
| MPV (MEDIAN [IQR]) | 10.70 [10.00, 11.40] | 10.60 [10.00, 11.35] | 0.505 | 0.055 |
| PDW (MEDIAN [IQR]) | 15.60 [12.80, 16.40] | 15.50 [12.50, 16.30] | 0.490 | 0.075 |
| PCT (MEDIAN [IQR]) | 0.24 [0.21, 0.28] | 0.25 [0.22, 0.28] | 0.198 | 0.061 |
| CIMT visit 1 (median [IQR]) | 0.75 [0.65, 0.80] | 0.65 [0.60, 0.75] | <0.001 | 0.509 |

**Note:** Continuous variables are presented as median (interquartile range, IQR), and categorical variables as n (%). Group comparisons were performed using the Mann-Whitney U test (for continuous variables) and Chi-square test or Fisher’s exact test (for categorical variables). CIMT = carotid intima-media thickness; BMI = body mass index; SBP = systolic blood pressure; DBP = diastolic blood pressure; TC = total cholesterol; TG = triglyceride; HDL = high-density lipoprotein cholesterol; LDL = low-density lipoprotein cholesterol; FBG = fasting blood glucose; ALT = alanine aminotransferase; TBIL = total bilirubin; TP = total protein; ALB = albumin. GLOB = globulin; BUN = urea nitrogen; CR = creatinine; UA = uric acid; WBC = white blood cell count; RBC = red blood cell count; HGB = hemoglobin; HCT = hematocrit; PLT = platelet count; MCV = mean corpuscular volume of erythrocytes; MCH = mean corpuscular hemoglobin content of erythrocytes; MCHC = mean corpuscular hemoglobin concentration of erythrocytes; RDW = Red Blood Cell Distribution Width; MPV = Mean Platelet Volume; PDW = Platelet Distribution Width; PCT = Platelet Compaction;SMD = standardized mean difference. SMD < 0.2 indicates a trivial difference, 0.2–0.5 a small difference, 0.5–0.8 a moderate difference, and >0.8 a large difference.
